# Improvement in Muscular Strength in HIV-Infected Individuals Receiving Antiretroviral Therapy

**DOI:** 10.3390/jfmk4030066

**Published:** 2019-09-14

**Authors:** Takshita Sookan, Ayesha Motala, Michael Ormsbee, Jose Antonio, Nombulelo Magula, Umesh Lalloo, Andrew McKune

**Affiliations:** 1 Biokinetics, Exercise and Leisure Sciences, University of KwaZulu-Natal College of Health Sciences, Durban 4000, KwaZulu Natal, South Africa; mormsbee@fsu.edu (M.O.); Andrew.McKune@canberra.edu.au (A.M.); 2 Department of Diabetes and Endocrinology, University of KwaZulu-Natal College of Health Sciences, Durban 4013, KwaZulu Natal, South Africa; MOTALA@ukzn.ac.za; 3 Department of Nutrition, Food, and Exercise Sciences, Florida State University, Tallahassee, FL 32306, USA; 4 Exercise and Sport Science, Nova Southeastern University, Davie FL 33328, USA; ja839@nova.edu; 5 Department of Internal Medicine Durban, University of KwaZulu-Natal College of Health Sciences, Durban 4013, KwaZulu Natal, South Africa; Magulan@ukzn.ac.za; 6 Department of Pulmonology Durban, University of KwaZulu-Natal College of Health Sciences, Durban 4013, KwaZulu Natal, South Africa; umeshlalloo@gmail.com; 7 Discipline of Sport and Exercise Science, University of Canberra, Canberra 2617, Australia

**Keywords:** resistance training, whey, placebo, detraining, HIV, exercise, ART

## Abstract

Purpose: This study investigated (1) the effect of a progressive resistance training (PRT) program and whey protein intake on maximal muscle strength in human immunodeficiency virus (HIV)-infected individuals receiving antiretroviral therapy (ART) and (2) alterations in maximal strength 12 wks after the cessation of PRT with continued supplementation. Methods: Sixty HIV-infected individuals were recruited. Whole body PRT was performed twice weekly for 12 wks. Participants received, in a double-blind placebo controlled manner, either 20 g whey or placebo (maltodextrin) before and immediately after each session. Both PRT groups continued to take either whey protein or placebo for a further 12 wks following the exercise intervention to examine the effects of detraining. Results: Forty participants (mean and standard deviation (SD) age 40.8 (±7.7) years, weight 70.8 (±16) kg, body mass index (BMI) 30.9 (±7.2) kg m^2^); whey protein /PRT (*n* = 13), placebo/PRT (*n* = 17), and a control group (*n* = 10) completed the study. A significant main effect for time occurred for the bench press (*p* = 0.02), the squat (*p* < 0.0001), the deadlift (*p* = 0.001) and the shoulder press (*p* = 0.02) one-repetition maximum (1RM) in the intervention groups. Conclusion: The PRT program increased maximal strength regardless of whey protein intake. The detraining period demonstrated minimal strength loss, which is beneficial to this population.

## 1. Introduction

The advancement of antiretroviral therapy (ART) for people with human immunodeficiency virus (HIV) has resulted in longer life expectancies and has converted HIV into a more manageable chronic disease [1,2,3]. However, this has resulted in health complications related to premature aging, including muscle wasting and other body composition changes [4,5]. These conditions increase the risk of functional decline, co-morbidities and associated mortality in HIV-infected individuals [6]. The loss of metabolically lean active tissue and malnutrition signify a poor prognosis for HIV-infected individuals [7,8]. HIV-related disability has also been associated with decreased exercise capacity and the impairment of patients’ daily activities [2,9,10].

Skeletal muscle represents between 50% and 54% of lean body mass in HIV-infected individuals, and muscle wasting is thus accompanied by decreased muscular strength and functional performance, as well as being associated with HIV disease progression [11,12,13]. The loss of lean body mass can occur independent of total body weight changes [11]. In an HIV infection, sacropenia can be demonstrated soon after HIV infection. This is important for two reasons: First, because there is a strong, direct relationship between muscle mass and strength, and, second, because muscle is the primary reservoir of amino acids for gluconeogenesis and protein synthesis in the muscles, the liver, and elsewhere [11,14,15]. In developed countries, the primary aim of therapy is to restore lean tissues, develop strategies to increase metabolically-active lean tissue, which is comprised primarily of muscle. These are all important goals in the management of persons infected with HIV [5,7].

In HIV-infected individuals, total protein catabolism is increased, releasing amino acids into systemic circulation from skeletal muscles. This breakdown provides energy substrates for other metabolically-active cells [7,16,17,18]. Muscle mass may also be affected by several anabolic hormones in HIV-infected individuals. For example, testosterone concentrations have been directly associated with muscle mass. Studies have found a loss of muscle mass, decreased CD4+ counts, an increased incidence of opportunistic infections, and wasting syndrome with low levels of testosterone., Skeletal muscle protein synthesis, which contributes to increased muscle mass, is regulated by testosterone [7,19,20]. 

Adequate muscle mass is crucial for functional health and quality of life. Research in older adults has found that an increase in muscle mass is shown to have a decrease in mortality risk in these individuals irrespective of fat mass, cardiovascular risk factors and metabolic risk factors Therefore, maintaining muscle mass or stimulating muscle hypertrophy is imperative. [10,21,22,23].

The most non-pharmacological efficient way to increase the size of a skeletal muscle is by resistance training (RT) in combination with protein-containing nutrition [18]. It has been clearly demonstrated that an acute bout of heavy resistance exercise (6–90% one-repetition maximum (1RM)) stimulates a significant increase in muscle protein synthesis [24]. Muscle hypertrophy due to RT and protein nutrition seems largely to result from cumulative acute increases in muscle protein synthesis. One RT bout can increase muscle protein synthesis within 1 h. Research has shown that the addition of protein intake pre and post RT can increase this response and contribute to muscle hypertrophy more than ingestion at other times of day [18,24]. 

Various types of proteins have been investigated in the literature in relation to RT and muscle protein synthesis that contribute to muscle hypertrophy. Research has identified whey protein to be the most suitable when combined with RT to stimulate muscle hypertrophy [18,24,25,26]. The ingestion of protein with carbohydrate or only branched chain amino acids in the context of a bout of RT has been shown to increase the phosphorylation of the mammalian target of rapamycin (mTOR) and p70S6K at 0–4 h post-RT in humans. Muscle protein synthesis and hypertrophy are stimulated by the mTOR pathway protein kinase enzymes [18,24,25,26]. 

The evidence available suggests that RT combined with a whey protein supplementation distal and proximal to the training session can increase muscle protein synthesis, aiding recovery and promoting muscle hypertrophy [25,27,28]. This type of intervention can play a vital role in an HIV-infected population in which premature aging, the wasting of metabolically-active tissue, and body fat redistribution are extensive [28,29]. The use of RT and whey has been used in the context of HIV-infected individuals; however, to our knowledge, there have not been any studies which have examined the intake of whey immediately before and after the training session. There are no studies which have reported on the effects of maximal strength following a 12 wk period of detraining with continued supplementation (whey protein or placebo) in HIV-infected individuals receiving ART.

Therefore, the aim of this study was to investigate (1) the effect of a progressive resistance training (PRT) program and whey protein intake on maximal muscle strength in HIV-infected individuals receiving antiretroviral therapy (ART) and (2) alterations in maximal strength 12 wks after the cessation of PRT with continued supplementation. We hypothesized that (1) the PRT and protein supplementation would increase muscle strength in HIV-infected patients when compared to PRT alone, and (2) protein supplementation would minimally alter maximal strength during the detraining period when compared to the placebo.

## 2. Materials and Methods 

### 2.1. Study Design and Participant Recruitment

In this randomized double-blind placebo controlled study, participants were recruited from the King Edward VIII Hospitals HIV Clinic in Durban, KwaZulu-Natal, South Africa. Sixty HIV-infected individuals adults (>18 years), with a CD 4+ T cell count of > 350 uL/mm were included in the study. They had to be on ART for a minimum of 18 months. Participants were excluded if they were participating in any purposeful exercise training (>30 min/day), pregnant or lactating, experienced a cold or feverish illness at least one month leading up to the protocol, and lastly, were lactose intolerant because whey protein is a dairy by-product. 

The participants were randomly assigned using the site research randomizer (www.randomizer.org, last accessed date: 14 January 2014) to one of three groups: A whey protein/progressive resistance training program (PRT) group, a placebo/PRT group, or a control group (received no exercise or supplementation). All participants were encouraged to maintain their normal activities and normal diet during the experimental period. The study was approved by the University of KwaZulu-Natal Biomedical Research Ethics Committee (BFC032/13). Participants signed an informed consent in either English or a translated isiZulu version for those that spoke isiZulu as a first language. Participants were given monetary assistance for transportation to attend all the testing and training sessions. 

Measurements including height, weight, waist to hip circumferences, 1RMs, and full blood count were done at baseline (pre), 12 wk (post intervention) and 6 months (detraining). The time (T) points (T1 = pre, T2 = 12 wks post intervention, T3 = 24 wks (detraining (T2–T3) = 3 months of supplementation but no exercise) are discussed in this paper.

### 2.2. Resistance Training Program

Progressive resistance training workouts were undertaken in the mornings twice a week for three months. Each session lasted ~60 min. During T1, 1RM testing was done together with other baseline measurements to determine each participant’s individual PRT program [30]. Resistance training was performed with progressive training loads of 40–85% of the participants’ 1RM using a periodized training program. Prior to the commencement of each session, the Wisconsin upper respiratory tract infection questionnaire (WURRS-21) was administered to ensure participants were well enough to train [31]. All training sessions were supervised by registered clinical exercise specialists. A brief warm-up, which included low intensity dynamic stretches, was done. The lower body exercises included the weighted squat, the bilateral lunge and the deadlift. The upper body exercises included the bench press, the shoulder press and the bicep curl, as well as abdominal/core exercises. Resistance training was performed with progressive training loads of 40–85% of the participants’ 1RM using a periodized training program. For each exercise in a workout, the number of sets increase (from 2–3 to 3–5), and the number of repetitions in each set decreased (from 15–20 to 5–6) during the 12 week resistance period. The loads were individually determined throughout the resistance training period. Recovery between the sets was 2–3 min. A short cool down was performed after the resistance training exercises, which included low intensity static stretches. No resistance training was performed in the control group. After the 12 wks of RT (T2), all participants were followed up for an additional 12 wks (post intervention –T3) to examine the detraining effect. 

### 2.3. Nutritional Supplementation 

Immediately before and after each PRT session, participants ingested either 20 g of whey protein (Vital Pharmaceuticals, Inc., Weston, FL, USA) dissolved in 250 mL of water or an equivalent dose of an isocaloric maltodextrin placebo. The drinks were provided to the participants in a double-blind fashion. Following the 12 wk (T2) PRT, participants were followed up for a further 12 wks (T3); during this period, they continued to take the supplementation they were allocated during the intervention (whey protein or placebo), but no exercise was done. Placebo and whey protein were prepacked in individual plastic bags. Participants were telephoned weekly by the research assistant to remind them to take the supplement twice a week. Participants were also interviewed at the 24 wk (T3) time point as to whether they took the supplement given to them.

### 2.4. Data Analysis

Data analyses was performed using the IBM SPSS Statistics for Windows software package, Version 23.3.1. IBM Corp., Chicago. The primary aim of the study was to look at whey/PRT compared to placebo/PRT. Therefore, changes in maximal strength over time and differences between the whey protein and placebo groups were analyzed using a two-way ANOVA with multiple comparisons (2 groups × 3 time points). Sidak’s multiple comparisons test was then performed to determine group, time and interaction effects. Changes in maximal strength over time in the control group were examined using a one-way ANOVA with repeated measures. Independent t-tests were performed to determine significant differences of baseline demographic data between the intervention groups and the control group. Cohen’s d effect sizes (ES) were also calculated and classified by Hopkins (2009) as: <0.2 (trivial), ≥0.2 to 0.59 (small), ≥0.6 to 1.19 (medium), and ≥1.2 (large). The alpha was set at *p* ≤ 0.05 and estimated 95% confidence intervals. 

## 3. Results

Out the 60 participants enrolled, 40 (66.7%) (80% female) completed the study (whey protein/PRT group (*n* = 13), placebo/PRT group (*n* = 17), and control (n = 10) group. Participants were excluded from analysis if they missed three consecutive training sessions, felt ill and were unable to attend, or had personal commitments that prevented them from continuing to attend sessions. Participants that completed the study complied with the requirements of the intervention and the detraining period. The mean age, weight and body mass index (BMI) of the 40 participants who completed the study are shown in Table 1. There was no significant difference in weight and BMI between the placebo/PRT group and the control group. A significant difference in weight (*p* = 0.02) and BMI (*p* = 0.03) between the whey/PRT group and the control group was noted. There was no significance between the groups for age and CD4+ T cell counts. 

**Figure 1:**
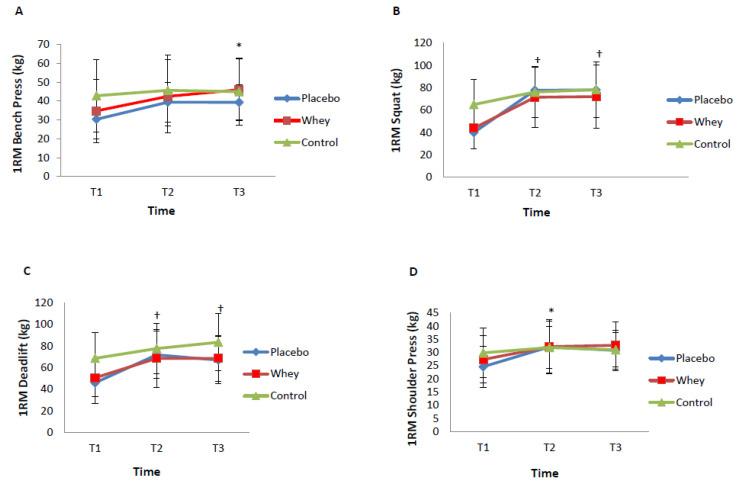
One-repetition maximum strength changes over time for the bench press (**A**), the squat (**B**), eth deadlift (**C**) and the shoulder press (**D**) from pre (T1); 12 wks post (T2); 24 wks post (T3) in the whey, placebo and control groups. Values are reported as means ± SD. Data were analysed using a two-way ANOVA with multiple comparisons (two groups x three time points) between the whey protein and placebo groups. The control group was examined using a one-way ANOVA with repeated measures. * *p* < 0.05, ^†^ *p* < 0.001, significant changes between T1, T2 and T3 between whey and placebo groups.

Table 2 shows the strength variables for PRT exercises in the whey/PRT and placebo/PRT groups over the three time points (T1; T2; T3). The significant main effects for time for the maximal strength variables are shown in Figure 1. In the placebo/PRT and whey/PRT groups, maximal strength increases were seen for the bench press (*p* = 0.02), the squat (*p* < 0.001), the deadlift (*p* < 0.001), the bicep curl (*p* = 0.04) and the shoulder press (*p* = 0.02). Figure 1 shows the significant time effects from pre (T1) and 12 wks post intervention (T2) and then from 12 wks (T2) to six months (T3) (the detraining period where no PRT was performed but supplementation continued). The upper body exercises of the bench press and the shoulder press increased in maximal strength from T1 to T3 in the placebo/PRT and whey/PRT groups. The lower body exercises of the squat and the deadlift increased in maximal strength from T1 to T2 and from T1 to T3 in the placebo/PRT and whey/PRT groups. Post hoc testing revealed no specific timepoint changes over the three time points for the bicep curl. For the control group, there were no significant strength changes over time in any of the muscle groups over the six months of the study. 

The results of the ES are shown in Table 3 for measurements from T1–T2, Table 4 (T1–T3) and Table 5 (T2–T3). Effect sizes were calculated for the bench press, the squat, the deadlift, the bicep curl, the shoulder press and right and left grip strength. Predominately, trivial-to-small effects were demonstrated for the control group in all three comparisons. 

Effect sizes for the changes in 1RM strength T1–T2 post intervention (Table 3) showed positive medium-to-large ES for lower body exercise in both the placebo/PRT and whey/PRT groups. Upper body exercises demonstrated positive ES, which ranged from trivial to medium. These positive ES (small to medium) changes indicated an increase in maximal strength for these exercises. The largest ES overall were for the squat in both the placebo/PRT group ((*p* ≤ 0.001), ES: d = 1.5; 95% CI 0.8, 2.3) and the whey/PRT group ((*p* ≤ 0.001), ES: d = 1.2; 95% CI 0.4, 2). The upper body exercises showed medium ES for the bench press in the placebo/PRT group ((*p* = 0.02), ES: d = 0.9; 95% CI −0.2, 1.6). The whey/PRT group showed small ES for the bench press ((*p* = 0.02), ES: d = 0.4; 95% CI −0.4, 1.2). The bicep curl in both the placebo/PRT group ((*p* = 0.04), ES: d = 0.6; 95% CI 0.0, 1.3) and the whey/PRT group ((*p* = 0.04), ES: d = 0.6; 95% CI −0.2, 1.4) had a medium ES. All other upper body exercises showed predominately trivial-to-small (ES: d = 0.1; 95% CI −0.6, 0.8; d = 0.5 95% CI −0.3, 1.3) effect sizes. 

Table 4 shows the changes in 1RM strength from T1 to T3. The ES were all positive changes, indicating an increase in maximal strength for these exercises. As for T1–T2 (Table 3) the largest ES were shown for lower body exercises, specifically the squat in both the placebo/PRT ((*p* ≤ 0.001), ES: d = 1.7; 95% CI 0.9, 2.5) and the whey/PRT ((*p* ≤ 0.001), ES: d = 1.2; 95% CI 0.3, 2.0) groups. All the upper body exercises showed medium ES except for grip strength. The largest ES for the upper body exercises were for the bench press in the placebo/PRT ((*p* = 0.02), ES: d = 0.9; 95% CI 0.1, 1.6) group. The whey/PRT group for the bench press also showed a medium effect size ((*p* = 0.02), ES: d = 0.7; 95% CI −0.1, 1.5).

The effect sizes for changes in 1RM strength for the period of detraining (T2–T3) are shown in Table 5. All exercises for the placebo/PRT group showed negative ES except for the squat, and this indicated a decrease in maximal strength in this group. This decrease in strength ranged from trivial-to-medium changes (ES: d = −0.6; 95% CI from −1.3 to –0.1; d = 0.0; 95% CI −0.7–0.7). All exercises for the whey/PRT group showed positive ES except for the deadlift and right/left grip strength, and this indicated a further increase in maximal strength in this group. These ES ranged from trivial to small (ES: d = 0.0; 95% CI −0.8, 0.8 to ES: d = 0.2; 95% CI −0.6, 1.0). Overall, the results show that PRT improved muscular strength from T1 to T2, and the PRT and whey protein groups maintained muscle strength during the period of detraining.

## 4. Discussion

In this study, we investigated the effects of a PRT program and whey protein supplementation on muscle strength indices in HIV-infected individuals receiving ART. Individuals that were randomized into either a placebo/PRT group or whey/PRT showed significant improvement in strength indices. 

The significant changes over time were observed for the bench press (T1–T3), the squat (T1–T2 and T1–T3), the deadlift (T1–T2 and T1–T3), the bicep curl, and the shoulder press (T1–T3) with no changes being observed in the control group. The overall maximal strength gains in this study are similar to previous studies of resistance training alone or in combination with aerobic training, which demonstrated increased strength and increased muscle mass among HIV-infected men and women [11,14,32,33]. The strength gains found in our study are similar to those found by Sakkas et al. [34], who demonstrated increased muscle strength in HIV-infected men; in that study, participants were randomly assigned to receive creatine monohydrate or placebo for 14 weeks, performed resistance exercises on a Hoist 5000 Multi-Gym Fitness System (San Diego, CA), and completed four sets of eight repetitions at 80% of 1RM during each session. The creatine monohydrate did not provide any added benefit. Dolan and colleagues [14] conducted a 16-week randomized intervention study of a supervised home-based PRT and aerobic exercise program in 40 HIV-infected women. The effects on strength were most significant in this study. Another study in Nigeria examined the effect of a 12-weeks combined aerobic and resistance training program on cardiopulmonary fitness and strength in an HIV-infected females. Significant strength increases were noted in the biceps curl, the lateral pull-down, the chest press, the leg extension and the hamstring curl, while cardiorespiratory fitness increased [35]. The results of our study provide further evidence of the beneficial effects of PRT on HIV-infected individuals. 

In this study, strength gains occurred irrespective of the intake of whey protein, indicating no additional benefit in this cohort; however, whey protein intake maintained strength over a 12 wk period of detraining. Though the use of whey protein immediately before and after a training session has been reported to aid muscle protein synthesis [25], it has not been investigated in HIV-infected individuals receiving ART. A study by Agin et al. (2001) investigated the effects of whey protein and resistance training in 30 malnourished HIV-infected women that were not on ART. The women were followed up for a six-week control period and then randomized to a whey protein group, a PRT group, or a combination of the two for 14 weeks. Both interventions increased body cell mass, but the whey protein promoted weight gain and PRT promoted muscle function [36]. The use of whey protein may aid and maintain musculoskeletal strength during a training period and during a period of no training or detraining [25,26]. Recovering strength and physical fitness is the major goal of exercise in HIV-infected patients with wasting syndrome. Therefore, a resistance training program alone or in combination with a protein containing supplementation could be the basis of an exercise prescription for this group. It should be progressive so that resistance is increased as the patient becomes stronger [19]. There have been a number of earlier, non-controlled studies that showed resistance alone or in combination with aerobic training improved body composition, strength, and fitness and were well tolerated in the HIV population [1,17,37,38]. In this study, the PRT program and whey protein supplementation was well tolerated, and there was a high adherence (66.7%) to the intervention. Therefore, the results suggest that resistance training has potential prophylactic benefits associated with increased lean body mass. Resistance training can be coupled with ART and nutritional counselling to alleviate some of the symptoms associated with the HIV infection [34,39,40]. Affordable and sustainable interventions are required, especially in resource limited Sub-Saharan Africa. A PRT program can serve as a valuable tool and has been shown to be beneficial irrespective of whey protein intake. Whey protein may be used to maintain strength during times when exercise is not possible, or it may augment the effects of exercise.

To our knowledge, this is the first study to examine the effects of a PRT and intake of whey protein immediately before and after each training session in an African HIV-infected population receiving ART. Additionally, this is also the first study to look at the effects of maximal strength following a 12 wk period of detraining with continued supplementation (whey protein or placebo) in HIV-infected individuals receiving ART.

### 4.1. Limitations

Firstly, all participants enrolled in this study were outpatients, and, due to the time demand of the intervention program, the total number of participants as well as participants in each of the groups had a total dropout of 33.3%. Secondly, the participants in this study were not fully representative of the general HIV-infected population receiving ART, as these participants were from one public government hospital and from a single ethnic group. Thirdly, socio-economic status was not fully investigated, and more information should be gathered regarding the socio-economic status of participants for future studies. Fourthly, due to the randomization process, the baseline characteristics between groups was not controlled for. Fifthly, training frequency was limited to twice weekly due to the logistics and financial constraints of the study itself. Lastly, the study design did not account for whey protein intake alone during the intervention phase, and the effect sizes presented in this paper will help power future studies that could include this as part of the study design.

### 4.2. Strengths

The present study contributes to the literature and is the first of its kind to investigate the effects of a PRT and whey protein supplementation on HIV-infected individuals receiving ART in South Africa. The study design was a randomized, double-blinded placebo controlled study. The PRT program was individualized for each participant based on their 1RM at baseline. The participants were also continually assessed throughout the duration of the intervention. Previous studies of resistance training among HIV-infected patients have been disproportionate with respect to the gender, favoring male cohorts. This study group consisted of 80% female participants. The period of detraining provides novel information regarding weight maintenance in this population. The significant findings of this study can advocate for future exercise intervention studies in an HIV-infected cohort receiving ART, and it highlights PRT as a positive sustainable intervention that can be carried out in resource limited areas.

## 5. Conclusions

In conclusion, this study indicates large strength gains in the intervention groups (placebo/PRT and whey/PRT) that were mostly maintained during the detraining period. The detraining period (T2–T3) demonstrated little-to-no strength loss, particularly in the whey/PRT group, which suggests that strength is maintained with the intake of whey protein following a PRT program for at least 12 wks. The control group showed little-to-no change over the study period. This suggests that a PRT program and whey protein supplementation may be potential resources to counteract premature aging (sarcopenia and dynapenia) muscle wasting that accompanies the side effects in an HIV population receiving ART. 

## Figures and Tables

**Table 1:** Baseline characteristics of human immunodeficiency virus (HIV)-infected participants on antiretroviral therapy (ART).

Variable	Placebo/PRT (*n* = 17)	Whey/PRT (*n* = 13)	Control (*n* = 10)
**Females**	14	11	5
**Males**	3	2	5
**Age (years)**	41.0 ± 8.1	40.8 ± 7.9	40.9 ± 7.0
**Weight (kg)**	73.7 ± 17.4	87.6 ± 14.9	72.9 ± 12.8
**BMI (kg/m^2^)** **CD4+ (cells/mm^3^)**	28.7 ± 6.9 445.82 ± 200.19	34.2 ± 6.3 396.92 ± 173.99	27.8 ± 7.0 383.80 ± 151.27

Values reported as mean ± SD. Placebo/PRT: Placebo and progressive resistance training; whey/PRT: Whey and progressive. Resistance training; BMI: Body mass index.

**Table 2:** Strength variables in the whey/PRT (progressive resistance training) and placebo/PRT groups.

Strength Exercise (kg)	T1		T2		T3	
	Whey/PRT (*n* = 13)	Placebo/PRT (*n* = 17)	Whey/PRT (*n* = 13)	Placebo/PRT (*n* = 17)	Whey/PRT (*n* = 12)	Placebo/PRT (*n* = 15)
**Bench Press**	34.80 ± 16.84 (27.15–42.45)	30.32 ± 10.41 (23.63–37.00)	42.50 ± 19.36 (34.85–50.15)	39.41 ± 10.55 (32.72–46.10)	46.04 ± 16.36 (38.08–54.00)	39.33 ± 9.23 (32.21–46.45)

**Squat**	43.88 ± 18.64 (30.04–57.73)	40.03 ± 17.17 (27.93–52.13)	71.46 ± 27.12 (57.62–85.30)	77.82 ± 30.34 (65.72–89.93)	72.08 ± 28.16 (57.68–86.49)	78.00 ± 26.44 (65.12–90.88)

**Deadlift**	50.50 ± 17.33 (38.71–62.29)	45.88 ± 18.98 (35.57–56.19)	68.46 ± 26.64 (56.67–80.25)	71.72 ± 21.74 (61.41–82.03)	68.33 ± 21.25 (56.06–80.61)	66.90 ± 21.67 (55.92–77.88)

**Bicep curl**	24.19 ± 7.65 (19.85–28.53)	23.71 ± 6.75 (19.91–27.50)	28.92 ± 9.01 (24.58–33.26)	28.56 ± 8.17 (24.76–32.35)	29.38 ± 9.18 (24.86–33.89)	27.63 ± 6.60 (23.59–31.67)

**Shoulder Press**	27.31 ± 8.85 (22.37–32.25)	24.59 ± 783 (20.27–28.91)	32.19 ± 10.34 (27.25–37.13)	32.12 ± 9.84 (27.80–36.44)	32.71 ± 9.01 (27.57–37.85)	30.73 ± 7.64 (26.14–35.33)

**Grip Strength (R)**	32.69 ± 12.45 (26.78–38.60)	34.44 ± 11.33 (29.27–39.61)	36.08 ± 9.47 (30.17–41.99)	36.53 ± 12.21 (31.36–41.70)	33.58 ± 8.50 (27.43–39.74)	32.07 ± 8.96 (26.56–37.57)
**Grip Strength (L)**	31.73 ± 12.58 (26.64–36.82)	31.00 ± 8.76 (26.55–35.45)	34.62 ± 9.01 (29.53–39.70)	33.53 ± 9.15 (29.08–37.98)	28.75 ± 6.86 (23.46–34.04)	28.13 ± 8.11 (23.40–32.87)

Values reported as mean ± SD (95% confidence interval). Whey/PRT: Whey and progressive resistance training, placebo/PRT: Placebo and progressive resistance training kg: Kilogram, R: Right, L: Left.

**Table 3:** Effect sizes (ES) of one-repetition maximum (1RM) strength indices from T1 to T2 in the placebo/PRT, whey/PRT, and control groups in HIV-infected individuals receiving ART (mean ± SD).

	Placebo/PRT					Whey/PRT					Control				
Strength Exercises (kg)	T1	T2	Cohen’s d	95% CI	ES	T1	T2	Cohen’s d	95% CI	ES	T1	T2	Cohen’s d	95% CI	ES
**Bench Press**	30.32 ± 10.41	39.41 ± 10.55	0.9	−0.2, 1.6	Medium	34.80 ± 16.84	42.50 ± 19.36	0.4	−0.4, 1.2	Small	42.85 ± 19.34	45.71 ± 18.86	0.1	−0.7, 1.0	Trivial
**Squat**	40.03 ± 17.17	77.82 ± 30.34	1.5	0.8, 2.3	Large	43.88 ± 18.64	71.46 ± 27.12	1.2	0.4, 2	Large	65.00 ± 22.36	76.20 ± 23.04	0.5	−0.4, 1.4	Small
**Deadlift**	45.88 ± 18.98	71.72 ± 21.74	1.3	0.5, 2	Large	50.50 ± 17.33	68.46 ± 26.64	0.8	0.0, 1.6	Medium	68.50 ± 24.04	77.50 ± 23.12	0.4	−0.5, 1.3	Small
**Bicep Curl**	23.71 ± 6.75	28.56 ± 8.17	0.6	−0.0, 1.3	Medium	24.19 ± 7.65	28.92 ± 9.01	0.6	−0.2, 1.4	Medium	31.50 ± 9.85	31.20 ± 8.62	−0.03	−0.9, 0.8	Trivial
**Shoulder Press**	24.59 ± 7.83	32.12 ± 9.84	0.3	−0.4, 1	Small	27.31 ± 8.95	32.19 ± 10.34	0.5	−0.3, 1.3	Small	29.85 ± 9.37	31.86 ± 7.87	0.2	−0.6, 1.1	Small
**Grip Strength (R)**	34.44 ± 11.33	36.53 ± 32.07	0.1	−0.6, 0.8	Trivial	32.69 ± 12.45	36.08 ± 9.47	0.3	−0.5, 1.1	Small	39.70 ± 16.91	37.00 ± 9.52	−0.2	−1.1, 0.7	Small
**Grip Strength (L)**	31.00 ± 8.76	33.53 ± 9.15	0.3	−0.4, 1	Small	31.73 ± 12.58	34.62 ± 9.01	0.3	−0.5, 1	Small	38.00 + 16.60	36.10 ± 9.40	−0.1	−1.0, 0.7	Trivial

**Table 4:** Effect sizes (ES) of one-repetition maximum (1RM) strength indices from T1 to T3 in the placebo/PRT, whey/PRT, and control in HIV-infected individuals receiving ART (Mean ± SD).

	Placebo/PRT					Whey/PRT					Control				
Strength Exercises (kg)	T1	T3	Cohen’s d	95% CI	ES	T1	T3	Cohen’s d	95% CI	ES	T1	T3	Cohen’s d	95% CI	ES
**Bench Press**	30.32 ± 10.41	39.33 ± 9.23	0.9	0.1, 1.6	Medium	34.80 ± 16.84	46.04 ± 16.36	0.7	−0.1, 1.5	Medium	42.85 ± 19.34	45.00 ± 17.68	0.1	−0.8, 1.0	Trivial
**Squat**	40.03 ± 17.17	78.00 ± 26.44	1.7	0.9, 2.5	Large	43.88 ± 18.64	72.08 ± 28.16	1.2	0.3, 2.0	Large	65.00 ± 22.36	78.33 ± 24.87	0.6	−0.4, 1.5	Medium
**Deadlift**	45.88 ± 18.98	66.90 ± 21.67	1.0	0.3, 1.8	Medium	50.50 ± 17.33	68.33 ± 21.25	0.9	0.1, 1.7	Medium	68.50 ± 24.04	83.33 ± 26.46	0.6	−0.3, 1.5	Medium
**Bicep Curl**	23.71 ± 6.75	27.63 ± 6.60	0.6	−0.1, 1.3	Medium	24.19 ± 7.65	29.38 ± 9.18	0.6	−0.2, 1.4	Medium	31.50 ± 9.85	31.11 ± 9.61	−0.0	−0.9, 0.9	Trivial
**Shoulder Press**	24.59 ± 7.83	30.73 ± 7.64	0.8	0.1, 1.5	Medium	27.31 ± 8.95	32.71 ± 9.01	0.6	−0.2, 1.4	Medium	29.85 ± 9.37	31.00 ± 6.46	0.1	−0.8, 1.0	Trivial
**Grip Strength (R)**	34.44 ± 11.33	32.07 ± 8.96	−0.2	−0.9, 0.5	Small	32.69 ± 12.45	33.58 ± 8.50	0.1	−0.7, 0.9	Trivial	39.70 ± 16.91	35.22 ± 10.24	−0.3	−1.2, 0.6	Small
**Grip Strength (L)**	31.00 ± 8.76	28.13 ± 8.11	−0.3	−1.0, 0.4	Small	31.73 ± 12.58	28.75 ± 6.86	−0.3	−1.1, 0.5	Small	38.00 ± 16.60	32.67 ± 10.68	−0.4	−1.3	Small

**Table 5:** Effect sizes (ES) of one-repetition maximum (1RM) strength indices from T2 to T3 in the placebo/PRT, whey/PRT, and control in HIV-infected individuals receiving ART (Mean ± SD).

	Placebo/PRT					Whey/PRT					Control				
Strength Exercises (kg)	T2	T3	Cohen’s d	95% CI	ES	T2	T3	Cohen’s d	95% CI	ES	T2	T3	Cohen’s d	95% CI	ES
**Bench Press**	39.41 ± 10.55	39.33 ± 9.23	−0.0	−0.7, 0.7	Trivial	42.50 ± 19.36	46.04 ± 16.36	0.2	−0.6, 1.0	Small	45.71 ± 18.86	45.00 ± 17.68	−0.0	−0.9, 0.9	Trivial
**Squat**	77.82 ± 30.34	78.00 ± 26.44	0.0	−0.7, 0.7	Trivial	71.46 ± 27.12	72.08 ± 28.16	0.0	−0.8, 0.8	Trivial	76.20 ± 23.04	78.33 ± 24.87	0.1	−0.8, 1.0	Trivial
**Deadlift**	71.72 ± 21.74	66.90 ± 21.67	−0.2	−0.9, 0.5	Small	68.46 ± 26.64	68.33 ± 21.25	−0.0	−0.8, 0.8	Trivial	77.50 ± 23.12	83.33 ± 26.46	0.2	−0.7, 1.1	Small
**Bicep Curl**	28.56 ± 8.17	27.63 ± 6.60	−0.1	−0.8, 0.6	Trivial	28.92 ± 9.01	29.38 ± 9.18	0.1	−0.7, 0.8	Trivial	31.20 ± 8.62	31.11 ± 9.61	−0.0	−1.0, 0.9	Trivial
**Shoulder Press**	32.12 ± 9.84	30.73 ± 7.64	−0.2	−0.9, 0.5	Small	32.19 ± 10.34	32.71 ± 9.01	0.1	−0.7, 0.8	Trivial	31.86 ± 7.87	31.00 ± 6.46	−0.1	−1.0, 0.8	Trivial
**Grip Strength (R)**	36.53 ± 12.21	32.07 ± 8.96	−0.4	−1.1, 0.3	Small	36.08 ± 9.47	33.58 ± 8.50	−0.3	−1.1, 0.5	Small	37.00 ± 9.52	35.22 ± 10.24	−0.2	−1.1, 0.7	Small
**Grip Strength (L)**	33.53 ± 9.15	28.13 ± 8.11	−0.6	−1.3, 0.1	Medium	34.62 ± 9.01	28.75 ± 6.86	−0.7	−1.5, 0.1	Medium	36.10 ± 9.40	32.67 ± 10.68	−0.3	−1.2, 0.6	Small
